# Phytohormone Response of Drought-Acclimated *Illicium difengpi* (Schisandraceae)

**DOI:** 10.3390/ijms242216443

**Published:** 2023-11-17

**Authors:** Chao Wu, Baoyu Liu, Xiujiao Zhang, Manlian Wang, Huiling Liang

**Affiliations:** Guangxi Key Laboratory of Plant Functional Phytochemicals and Sustainable Utilization, Guangxi Institute of Botany, Guangxi Zhuang Autonomous Region and Chinese Academy of Sciences, Guilin 541006, China; lby@gxib.cn (B.L.); zxj@gxib.cn (X.Z.); wml@gxib.cn (M.W.); lhl@gxib.cn (H.L.)

**Keywords:** abscisic acid, cytokinins, medicinal plant, methyl jasmonate, physiology

## Abstract

*Illicium difengpi* (Schisandraceae), which is an endemic, medicinal, and endangered species found in small and isolated populations that inhabit karst mountain areas, has evolved strategies to adapt to arid environments and is thus an excellent material for exploring the mechanisms of tolerance to severe drought. In experiment I, *I. difengpi* plants were subjected to three soil watering treatments (CK, well-watered treatment at 50% of the dry soil weight for 18 days; DS, drought stress treatment at 10% of the dry soil weight for 18 days; DS-R, drought-rehydration treatment at 10% of the dry soil weight for 15 days followed by rewatering to 50% of the dry soil weight for another 3 days). The effects of the drought and rehydration treatments on leaf succulence, phytohormones, and phytohormonal signal transduction in *I. difengpi* plants were investigated. In experiment II, exogenous abscisic acid (ABA, 60 mg L^−1^) and zeatin riboside (ZR, 60 mg L^−1^) were sprayed onto DS-treated plants to verify the roles of exogenous phytohormones in alleviating drought injury. Leaf succulence showed marked changes in response to the DS and DS-R treatments. The relative concentrations of ABA, methyl jasmonate (MeJA), salicylic acid glucoside (SAG), and cis-zeatin riboside (cZR) were highly correlated with relative leaf succulence. The leaf succulence of drought-treated *I. difengpi* plants recovered to that observed with the CK treatment after exogenous application of ABA or ZR. Differentially expressed genes involved in biosynthesis and signal transduction of phytohormones (ABA and JA) in response to drought stress were identified by transcriptomic profiling. The current study suggested that the phytohormones ABA, JA, and ZR may play important roles in the response to severe drought and provides a preliminary understanding of the physiological mechanisms involved in phytohormonal regulation in *I. difengpi*, an endemic, medicinal, and highly drought-tolerant plant found in extremely small populations in the karst region of South China.

## 1. Introduction

The habitats of karst regions, which are characterized by a thin soil layer, intermittent drought, and alternation between wet and dry conditions, nurture a variety of plant species, including large proportions of most-at-risk species, such as plant species with extremely small populations [1]. Prolonged droughts cause more frequent occurrences of severe drought events, which increase the risk of plant withering and could lead to the extinction of extremely small plant populations; these effects thus threaten biodiversity conservation, ecological restoration, and agricultural production in karst regions [2]. Therefore, drought is a primary stress factor that needs to be urgently addressed. Karst habitats are expected to worsen with future climate changes. However, the understanding of the responses of endemic plants in karst habitats to drought stress and the underlying mechanisms is limited [3].

The karst landscape of South China represents the largest distinctive region of the three major karst areas in the world and is characterized by its unique geographical features, significant biological diversity, and large proportion of endemic species [4]. The plants in karst regions develop special adaptability to unique karst ecosystems with conspicuous landscape features [5]. *Illicium difengpi* (Schisandraceae) is an endemic plant found in the karst region of South China [3]. According to our previous investigation, *I. difengpi* plants are primarily distributed in the karst mountains of Guangxi, Yunnan, and Guangdong provinces in China [6]. In the wild, *I. difengpi* plants grow in bare and semi-bare patches of rocky mountain areas and thus demonstrate a typical characteristic of drought resistance [7]. In traditional Chinese medicine, the barks of the stem and root of *I. difengpi* are used to treat traumatic injury and rheumatoid arthritis [8]. However, wild resources of *I. difengpi* have declined sharply due to deforestation and over-excavation in recent years [3], which renders these plants an endangered species.

As an endemic, medicinal, and endangered species found in small and isolated populations in the karst region of South China, *I. difengpi* can adapt to and survive under severe drought stress and is thus regarded as an excellent material for studying plant tolerance to severe drought [3]. To cope with drought stress, plants undergo various physiological and biochemical changes, such as changes in water physiology, phytohormone regulation, and related gene expression [9]. Our previous studies showed that phytohormone signals are involved in the drought responses of *I. difengpi* plants [10]. Abscisic acid (ABA), salicylic acid (SA), cytokinin (CTK), ethylene (ET), indole-3-acetic acid (IAA), jasmonic acid (JA), gibberellins (GA), and brassinosteroids (BR) are important phytohormones for overcoming the challenges due to drought stress faced by higher plants [11]. However, the specific types of phytohormones and their regulatory mechanisms underlying drought tolerance in *I. difengpi* plants are unclear.

Leaf succulence has been proposed as a rapidly measurable and physiologically meaningful metric of plant water storage [12] and can be engineered to improve water-deficit stress attenuation and abiotic stress tolerance [13]. In the current study, the effects of drought and rehydration on leaf succulence and phytohormones of *I. difengpi* plants were investigated. The regulatory roles of targeted phytohormones that are strongly correlated with leaf succulence were partially validated by the exogenous application of related phytohormones. A transcriptome profiling of phytohormonal signal transduction in response to drought and rehydration was also conducted. The objective was to preliminarily reveal the physiological mechanisms of drought tolerance in *I. difengpi* plants in terms of phytohormonal regulation.

## 2. Results

### 2.1. Effects of Drought and Rehydration Treatments on Leaf Succulence

**Figure 1:**
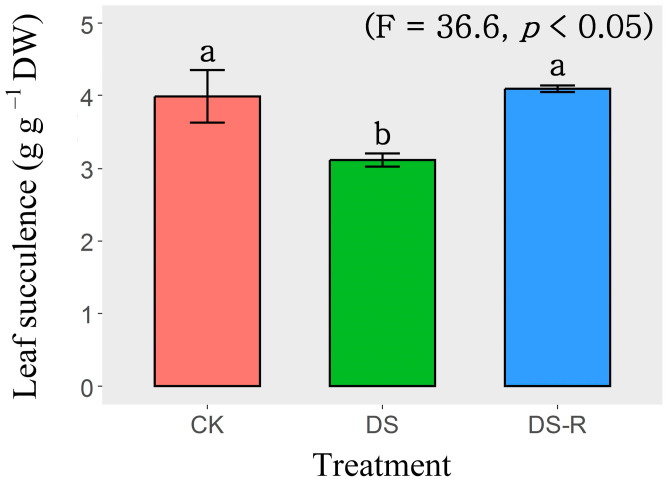
Effects of water treatments on leaf succulence. Data are presented as mean ± SD (n = 4). Different letters indicate significant differences among the three water treatments at the *p* < 0.05 level by a least significant difference test. CK, well-watered treatment; DS, drought stress treatment; DS-R, drought-rehydration treatment.

Variations in leaf succulence were observed among the three watering treatments (well-watered treatment, CK; drought stress treatment, DS; drought-rehydration treatment, DS-R). The DS treatment significantly (*p* < 0.05) reduced the leaf succulence of *I. difengpi* plants by 21.7%, compared with that of the CK-treated plants. The DS-R treatment significantly (*p* < 0.05) increased the leaf succulence of *I. difengpi* plants by 31.4% compared with that of the DS-treated plants. Notably, nonsignificant variations in leaf succulence were observed between the CK and DS-R treatments (Figure 1).

### 2.2. Effects of Drought and Rehydration Treatments on Phytohormones

The concentrations of phytohormones were significantly affected by the watering treatments. Compared with those obtained with the CK treatment, the concentrations of ABA and 1-aminocyclopropanecarboxylic acid (ACC) were increased significantly by the DS treatment; however, the DS-R treatment significantly increased the ABA concentration but negligibly affected the ACC concentration. The concentrations of methyl jasmonate (MeJA), salicylic acid glucoside (SAG), cis-zeatin (cZ), cis-zeatin riboside (cZR), and isopentenyl adenosine (iPA) were significantly decreased by the DS treatment, but the DS-R treatment significantly decreased the MeJA and iPA concentrations and significantly decreased the cZ and cZR concentrations. The concentration of N6-isopentenyladenine (iP) was decreased significantly by the DS-R treatment but was not affected by the DS treatment (Table 1).

### 2.3. Relationships between Phytohormones and Leaf Succulence under Watering Treatments

**Figure 2:**
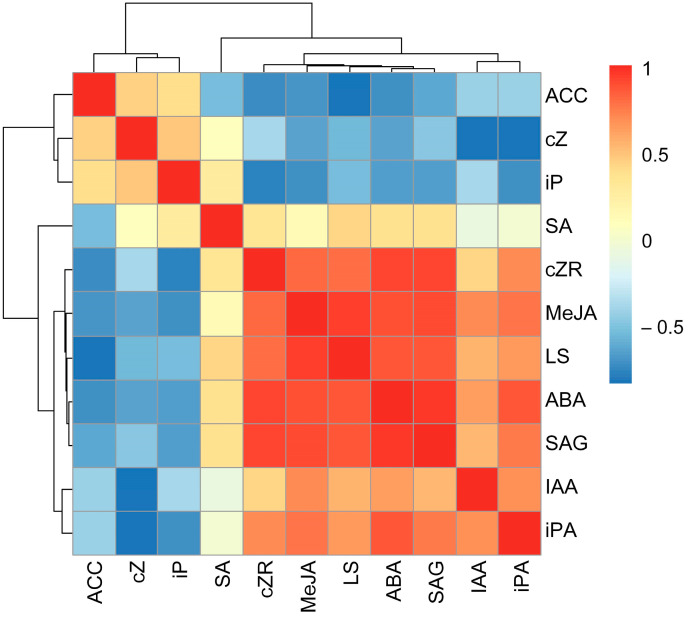
Relationships between relative values of phytohormone concentrations and leaf succulence under drought and rehydration treatments. LS, leaf succulence; ABA, abscisic acid; MeJA, methyl jasmonate; ACC, 1-Aminocyclopropanecarboxylic acid; IAA, indole-3-acetic acid; SAG, salicylic acid glucoside; SA, salicylic acid; iP, N6-isopentenyladenine; cZ, cis-zeatin; cZR, cis-zeatin riboside; iPA, isopentenyl adenosine.

The relative concentrations of MeJA (r = 0.93, *p* < 0.01), ABA (r = 0.86, *p* < 0.01), SAG (r = 0.87, *p* < 0.01), and cZR (r = 0.79, *p* < 0.05) were significantly positively correlated with relative leaf succulence, and the relative concentration of ACC was negatively correlated with relative leaf succulence (r = −0.84, *p* < 0.01). Nonsignificant correlations were detected between the relative concentrations of IAA, SA, cZ, iP, and iPA and leaf succulence in *I. difengpi* plants under drought and rehydration conditions (Figure 2).

### 2.4. Effects of Exogenous Phytohormone Application on Drought Stress Attenuation

**Figure 3:**
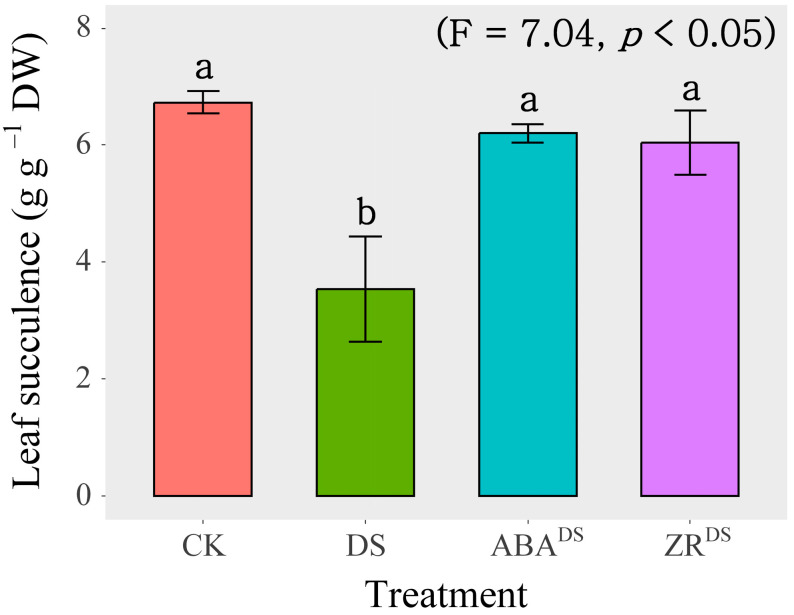
Effects of exogenous phytohormone application on leaf succulence under drought stress treatment. Data are presented as mean ± SD (n = 4). Different letters indicate significant differences among the three water treatments at the *p* < 0.05 level by a least significant difference test. CK, well-watered treatment; DS, drought stress treatment; ABA^DS^, exogenous ABA application under drought stress treatment; ZR^DS^, exogenous ZR application under drought stress treatment.

As shown in Figure 3, leaf succulence was decreased significantly by the DS treatment. Exogenous ABA application significantly increased leaf succulence by 75.8% compared with that obtained after dd-H_2_O spraying under drought conditions. The relative leaf succulence of ABA-treated plants under DS conditions recovered to the levels observed in the CK group. Exogenous ZR application significantly increased leaf succulence by 71.1% compared with that obtained with dd-H_2_O spraying under drought conditions. The relative leaf succulence of ZR-treated plants under DS conditions recovered to the levels observed in CK-treated plants.

### 2.5. Response of Key Unigenes Involved in Biosynthesis and Signal Transduction of Phytohormones

**Figure 4:**
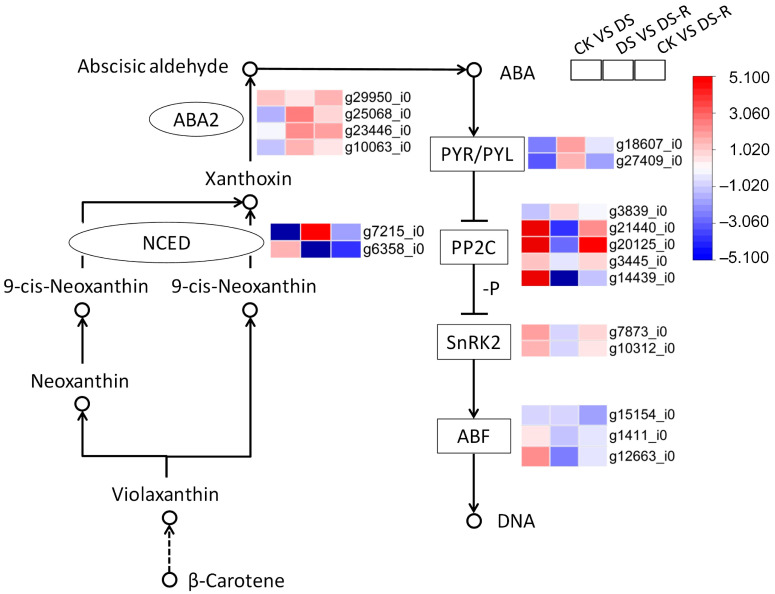
Differentially expressed genes involved in ABA signal transduction pathway under water treatments. ABA, abscisic acid; NCED, 9-cis-epoxycarotenoid dioxygenase; ABA2, Xanthoxin dehydrogenase, PYR/PYL, pyrabactin resistance 1-like protein; PP2C, protein phosphatases type 2C; SnRK2, sucrose non-fermenting-1-related protein kinase 2; ABF, ABRE-binding factor; DNA, deoxyribonucleic acid; CK, well-watered treatment; DS, drought stress treatment; DS-R, drought-rehydration treatment. Solid arrows indicate molecular interaction; dashed arrows indicate indirect link.

**Figure 5:**
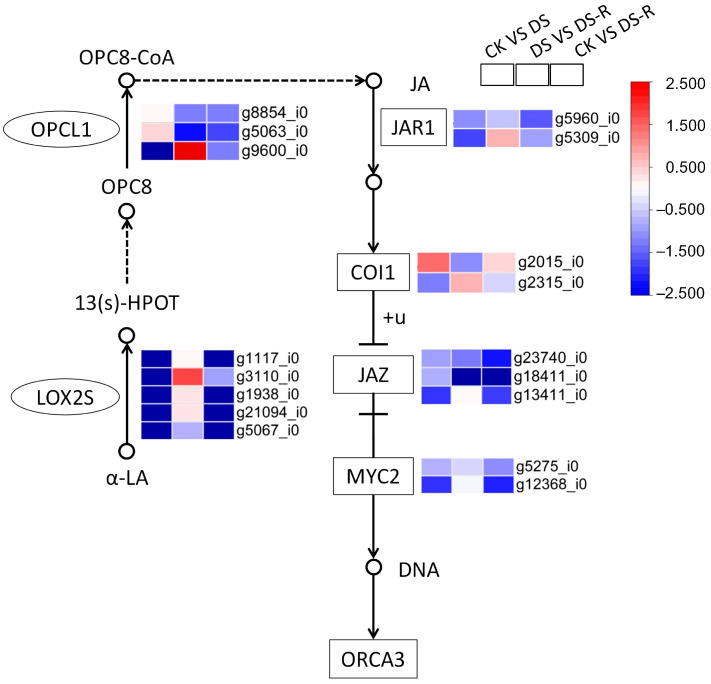
Differentially expressed genes involved in JA signal transduction pathway under water treatments. JA, jasmonate acid; LOX2S, lipoxygenase; OPCL1, OPC-8:CoA ligase 1; OPC8, cyclopentane-1-octanoic acid; 13 (S)-HPOT, 13-hydroperoxy-9,11,15-octadecatrienoic acid; α-LA, α-linolenic acid; OPC8-CoA, 3-oxo-2-(2′-pentenyl)-cyclopentane-1-hexanoic acid 8-CoA; JAR1, jasmonoyl amino acid conjugate synthase; COI1, F-box protein coronatine-insensitive 1; JAZ, Jasmonate ZIM-domain; MYC2, basic helix–loop–helix transcription factor; DNA, deoxyribonucleic acid; ORCA3, jasmonic acid-responsive AP2/ERF transcription factor; CK, well-watered treatment; DS, drought stress treatment; DS-R, drought-rehydration treatment. Solid arrows indicate molecular interaction; dashed arrows indicate indirect link.

Based on the results from the correlation analysis (Figure 2) and validation using exogenous phytohormone application (Figure 3), we emphasized the specific expression patterns of the ABA and JA signaling pathways (Figure 4 and Figure 5). DS treatment downregulated the expression of the ABA biosynthesis gene (*NCED*, *ABA2*), as well as that of the ABA receptor PYR/PYL family (PYL), and upregulated protein phosphatase 2C (PP2C) and serine/threonine-protein kinase SRK2 (SNRK2), which are involved in ABA signal transduction, compared with CK treatment. DS-R treatment unregulated *ABA2* and *NCED* gene as well as PYL, but downregulated the ABA receptors (PYL, SnRKS, and ABF), compared with DS treatment. The expression of *ABA2* gene, as well as PP2C and SnRK2, was upregulated higher than the control levels (Figure 4).

Generally, DS treatment downregulated the expression of lipoxygenase genes (*LOX*) and OPC-8:0 CoA ligase (*OPCL*) gene, which encode precursor molecules and intermediates of JA, respectively, as well as the expression of JA signal transduction genes, including jasmonic acid-amino synthetase (*JAR1*), jasmonate ZIM domain-containing protein (*JAZ*), and transcription factor MYC2, when compared with CK treatment. DS treatment downregulated the expression of JA biosynthesis- and signal transduction-related genes consistently and upregulated the *LOX*, *JAR1*, and *COI1* genes compared with CK treatment and DS treatment, respectively. The expression of coronatine-insensitive protein 1 (*COI1*) was regulated to become upregulated/downregulated, indicating the absence of JA response gene expression (Figure 5).

## 3. Discussion

Plant tissue succulence has been proposed as the direct metric of plant water storage and has thus been used as a drought tolerance index for the screening of drought-resistant genotypes of various species [12,14]. Numerous studies have found substantial decreases in plant tissue succulence under drought conditions, and even mild drought treatment can lead to significant reductions in leaf succulence/water content in various plant species [13,15,16]. In the present study, significant reductions in leaf succulence were induced by drought treatment (10% of the dry soil weight, which can be defined as severe drought stress when referring to many other species) compared with the control watering treatment (50% of the dry soil weight) (Figure 1). Leaf succulence was thus utilized to explore the physiological and molecular mechanisms underlying the responses of *I. difengpi* plants to drought in the current study.

The drought stress response is essentially driven by phytohormones and their intricate network of crosstalk, which leads to transcriptional reprogramming [17]. The current study revealed that changes in the ABA, MeJA, SAG, and cZR concentrations were strongly correlated with changes in leaf succulence under drought and rehydration conditions (Figure 2). Leaf succulence recovered to the CK level after exogenous application of ABA or ZR under drought conditions (Figure 3). Previously, ABA, JA, CTK, and SA are involved in drought tolerance in crops [18,19,20]. Taken together, our results showed that the phytohormones ABA and CTK regulate drought responses in endemic and medicinal *I. difengpi* plants, which show high tolerance to drought and are found in extremely small populations in the karst region of South China. However, the regulatory role of SA in the drought responses of *I. difengpi* plants needs further verification.

As an important signaling molecule in the response to drought stress, ABA triggers stomatal closure and osmotic regulation and regulates the expression of genes that confer tolerance to low water potential [21,22]. ABA biosynthesis and signal transduction are closely related to the drought resistance mechanisms of plants [23]. NCED is crucial to ABA synthesis, and its upregulation accelerates ABA production under drought stress [24]. In soybean, drought tolerance has been improved by overexpression of the *NCED* gene under controlled and open-field conditions [25]. In the current study, drought treatment induced upregulation of *NCED* gene expression and a significant increase in ABA accumulation in highly tolerant *I. difengpi* plants (Figure 4). Thus, the induction of ABA accumulation by drought due to enhanced expression of biosynthesis genes, such as *NCED*, may contribute to the drought tolerance of *I. difengpi* plants.

Increases in the intracellular ABA levels cause the ABA receptor PYL/PYR/RCAR proteins to bind to PP2C and release SnRK2s [22]. SnRK2s phosphorylate downstream substrates and initiate ABA responses [26]. Major SnRK2 protein kinases are responsible for ABA signal transduction. In the present study, significant differences were observed in the expression of four key genes (*PYL*, *PP2C*, *SNRKs*, and *ABF*) related to ABA signal transduction (Figure 4). The genes encoding PYL and PP2C were upregulated and downregulated, respectively, which is in accordance with the results found in *Sophora davidii* [27]. Plant SNRKs phosphorylate the NADPH oxidase RbohD, which results in the activation of Ca^2+^ signal transduction, the regulation of stomatal movement, and the combating of drought stress [22,26,28]. ABF is a TF belonging to the major bZIP family that regulates the expression of ABA-related genes under abiotic and osmotic stress conditions [29]. Moreover, an increase in the ABA content induces upregulated expression of numerous TFs and genes and thereby activates downstream metabolic pathways. In addition, ABA signal transduction weakened to levels close to those observed in the control group after rehydration, indicating drought stress attenuation (Figure 4). In summary, the existing results indicate that the expression of key genes involved in ABA accumulation is upregulated in *I. difengpi* under drought conditions and that this upregulation induces ABA signal transduction to regulate stomatal closure, maintain the water content, and enhance tolerance to drought stress.

Drought stress stimulates JA signaling, which mediates plant stress responses. The current study found that drought treatment generally decreased the expression of *LOX* genes encoding precursor molecules of JA and significantly increased the expression level of the *OPCL1* gene encoding an intermediate of JA, and the opposite trend was observed under rehydration treatment (Figure 5). Various studies have suggested that the JA biosynthetic genes *LOX* and *OPCL1* are involved in the response to abiotic stresses, including drought, by regulating JA synthesis in plants; for example, the expression of *OPCL1* is increased under drought in tea plants [30], and overexpression of *CmLOX10* enhances drought tolerance by promoting JA accumulation and stomatal closure in oriental melon [31]. In fact, JA and ABA coordinate to regulate each other’s responses to water treatments [32], and studies have shown that JA acts upstream of ABA by drought priming in wheat [33]. As discussed above, it was speculated that JA synthesis may play an important role in integrating the responses of *I. difengpi* plants to drought. However, the downregulated genes related to JA signal transduction (*JAZ*, *JAR1*, and *MYC2*) under both drought and rehydration treatments (Figure 5) indicate that the JA signaling pathway in *I. difengpi* plants remains inactive. Overall, we hypothesize that ABA and JA synthesis is essential to drought tolerance in *I. difengpi* plants.

## 4. Materials and Methods

### 4.1. Experiment I: Physiological and Transcriptomic Responses to Drought

#### 4.1.1. Plant Cultivation

During an investigation of wild medicinal plant resources, a single wild *I. difengpi* tree native to the top of a karst mountain (23°13′ N, 106°1′ E, 1130 m) in Jingxi City, Guangxi Province, was labeled and has been under long-term observation since 2011 [6]. In the current study, *I. difengpi* plants were produced from seeds collected from the labeled *I. difengpi* tree. The seeds of *I. difengpi* were soaked for germination in November 2019 at the Guangxi Institute of Botany, Chinese Academy of Science, Guilin City, Guangxi Province, China (25°4′ N, 110°18′ E). After soaking, the sprouted seeds were sown on a sand bed for seedling establishment in a plastic greenhouse. The *I. difengpi* seedlings were well cultivated and properly spaced, and regular watering and weeding were conducted to obtain robust and consistent *I. difengpi* plants in an open-net shed covered with a shading net.

**Figure 6:**
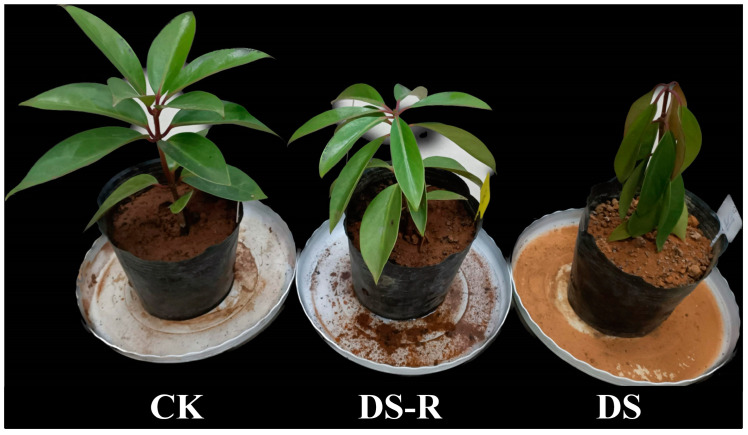
Images showing *I. difengpi* plants under water treatments. CK, well-watered treatment; DS, drought stress treatment; DS-R, drought-rehydration treatment.

Clay soil was crushed and dried to a constant weight at 80 °C in an oven for 3 days. The dried soil was weighed and placed in black nutritional bowls (height, 5.80 cm; top diameter, 5.80 cm; base diameter, 3.75 cm). Each nutritional bowl contained a mixture of 1.35 kg of dried soil and 1.0 g of urea. All nutritional bowls were thoroughly watered, and the saturated soil water content (0.61 ± 0.008 kg) of each nutritional bowl was calculated. Twelve two-year-old *I. difengpi* plants of uniform heights (22~24 cm) and similar numbers of leaves (12~14 leaves) and the same fresh weight (0.05 ± 0.004 kg) were excavated carefully to avoid root damage. The *I. difengpi* plants were transplanted into nutritional bowls and cultivated for 15 days, and during this watering period, the soil moisture of each bowl was monitored every day in real time by weighing the total weight of the bowl using a balance. The selected *I. difengpi* plants were subjected to the watering treatments on 25 February 2022 (Figure 6).

#### 4.1.2. Water Treatments

We previously evaluated the growth and physiological responses of *I. difengpi* plants to a series of moisture gradients (saturated soil water contents of 70~80%, 50~60%, 30~40%, 20~30%, and 10~20%) and rewatering treatments and found that *I. difengpi* plants can survive under severe drought conditions consisting of a saturated soil water content of 10~20%, and significant variations were observed between a saturated soil water content of 10~20% and the other water regimes; otherwise, *I. difengpi* plants appear to not have the ability to adapt to excessive moisture conditions consisting of a saturated soil water content of 70~80%. In the present study, *I. difengpi* plants were subjected to three soil watering treatments, namely, a well-watered treatment at 50% of the dry soil weight for 18 days (CK), drought stress treatment at 10% of the dry soil weight for 18 days (DS), and drought-rehydration treatment at 10% of the dry soil weight for 15 days followed by rewatering to 50% of the dry soil weight for another 3 days (DS-R). Four independent biological replicates of each group were included.

The soil moisture of the water treatments In the present study was controlled according to the dry soil weight (1.35 kg). Accordingly, watering with 0.675 kg of H_2_O and 0.135 kg of H_2_O was performed to achieve the target soil moistures of 50% and 10% under the CK and DS treatments, respectively. In addition, watering with 0.135 kg of H_2_O and 0.675 kg of H_2_O was applied during days 0~15 and days 16~18, respectively, under the DS-R treatment. We monitored the weight of each bowl 3 times (in the morning, at noon, and at sunset) every day and controlled the soil moisture precisely by watering the bowls to the target weight (Table 2). The plants were grown in a plastic greenhouse during the watering treatment to avoid rain interference. After the watering treatments, fresh leaf samples (from the 2nd to 4th mature functional leaves from the top) were collected, flash-frozen in liquid nitrogen for 30 s, and stored at −80 °C for analyses of enzyme activity, phytohormones, and transcriptome profiles.

### 4.2. Experiment II: Verification of Exogenous Phytohormone Effects on Drought Stress Attenuation

One-year-old uniform *I. difengpi* plants were selected to verify the roles of exogenous phytohormones (ABA and ZR) in alleviating drought injury. In this experiment, two watering treatments, CK (50% of the dry soil weight) and DS (10% of the dry soil weight), were included. Two exogenous phytohormones, ABA (60 mg L^−1^, Sigma–Aldrich, St. Louis, MO, USA) and ZR (60 mg L^−1^, Sigma–Aldrich, St. Louis, MO, USA), were mixed with two drops of 0.01% Tween 20 and sprayed onto the DS-treated plants. The application of exogenous phytohormones was conducted twice at dose of 5 mL plant^−1^ on the first day and second day after drought treatment began. Plastic film was hung between the treated and untreated groups to avoid ABA/ZR drifting onto the untreated plants. The same volume of double-distilled H_2_O (dd-H_2_O) was sprayed onto the control plants in the DS-treated group. Leaves were sampled for measurement after 15 days of treatment.

### 4.3. Leaf Succulence

Leaf succulence was used to indicate the leaf water status of *I. difengpi* plants. The fresh leaf mass (g) was determined immediately after detachment. The leaves were then incubated with distilled water for 3 days to measure the saturated water content after full hydration. The dry leaf mass (g) was then determined after oven-drying at 80 °C to a constant weight. Leaf succulence was calculated as follows: leaf succulence = [fresh leaf mass (g) − dry leaf weight (g)]/dry leaf mass (g).

### 4.4. Phytohormone Analysis

The leaf samples were ground to a fine power in liquid nitrogen using a mixer mill (Tissuelyser-48, Shanghai Jingxin Industrial Development Co., Ltd., Shanghai, China). Fifty milligrams of powder was weighed into a 1.0-mL tube, mixed with 0.5 mL of cold extraction buffer (acetonitrile, Fischer Scientific Co., Ltd., Waltham, USA), shaken on a shaking bed for 12 h at 4 °C, and then centrifuged at 12,000 rpm for 10 min at 4 °C. The supernatant was transferred to a new centrifuge tube. The pellets were extracted twice by exposure to 0.5 mL of acetonitrile for 4 h. All supernatants were pooled and purified using a C_18_-SepPak cartridge (Waters Corporation, Milford, MA, USA). The purified samples were freeze-dried by evaporation and then dissolved in 200 µL of acetonitrile solution (10%) for phytohormone determination.

The sample extracts were analyzed using a liquid chromatography–electrospray ionization–tandem mass spectrometry (LC–ESI–MS/MS) system (HPLC, Shim-pack UFLC SHI-MADZU CBM30A system, Shimadzu Corporation, Kyoto, Japan; MS, Applied Biosystems 4500 QTRAP, AB SCIEX Pte. Ltd., Framingham, MA, USA) equipped with a Waters ACQUITY UPLC HSS T3 column (2.1 × 100 cm, 1.8 μm; Waters Corporation, Milford, MA, USA) by multistep linear gradient elution (14 min) at a flow rate of 0.4 mL min^−1^ under a constant column temperature of 40 °C. The elution gradient was achieved with a binary solvent system consisting of 0.1% formic acid (Fisher, Fair Lawn, NJ, USA) in dd-H_2_O (solvent A) and 0.1% formic acid in acetonitrile (solvent B). The following protocol was used for gradient elution: 0 min, 95% A and 5% B; 10 min, 5% A and 95% B; 11 min, 5% A and 95% B; 11.1 min, 95% A and 5% B; and 14 min, 95% A and 5% B.

Linear ion trap (LIT) and triple quadrupole (QQQ) scans were acquired with a QQQ–LIT mass spectrometer (QTRAP) API 4500 QTRAP LC–MS/MS System equipped with an ESI Turbo Ion-Spray interface, which was operated in the positive ion mode and controlled by Analyst 1.6 software (AB Sciex, Framingham, MA, USA). The ESI source operation parameters were as follows: ion source, turbo spray; source temperature, 550 °C; ion spray voltage (IS), 5500 V; ion source gas I (GSI), gas II (GSII), and curtain gas (CUR), 55, 60, and 35.0 psi, respectively; and collision gas (CAD). The DP and CE for individual MRM transitions were performed with further DP and CE optimization. A specific set of MRM transitions was monitored during each period according to the metabolites eluted within the period.

### 4.5. Transcriptome Analyses

Total RNA was extracted from triplicate samples of leaves of the DS-, DS-R-, and CK-treated *I. difengpi* plants. The methods of RNA extraction, cDNA preparation, and Illumina sequencing were performed as described previously [10], and sequencing was conducted on the DNBSEQ-T7 platform using the PE150 strategy. The library was subjected to paired-end 150-bp sequencing using the NovaSeq 6000 system (Illumina, San Diego, CA, USA) if the quality standards were met. The raw data have been uploaded to the National Center for Biotechnology Information (NCBI) Short Read Archive (SRA) database under BioProject number PRJNA983054.

We determined the sequencing error rate, calculated the GC content distribution, removed low-quality sequences, and removed contaminating adaptors to obtain clean reads. The filtered sequences were aligned to the reference genome using HISAT2. All genes were annotated by BLAST using the Kyoto Encyclopedia of Genes and Genomes, NCBI nonredundant protein sequences, Swiss-Prot, Gene Ontology, Clusters of Orthologous Groups, and TrEMBL databases under the guidance of experts at Norminkoda Biotechnology Co., Ltd. (Wuhan, Hubei, China).

Fragments per kilobase of transcript per million fragments mapped was used as an indicator of gene expression levels. These data were then used to determine the overall distribution of sample gene expression. Heatmaps were prepared using TBtools. The Benjamini–Hochberg correction method was used, the resulting significant *p* values were corrected, and padj was used as a key indicator for screening genes with differential expression. Differentially expressed transcripts/genes were screened using DESeq2 with the criteria of fold change ≥ 2 and padj < 0.05. The Plant Transcription Factor Database was used to identify the transcription factors.

Seven differentially expressed genes were subjected to qRT–PCR assays to verify the accuracy of the RNA-seq results of *I. difengpi* under watering treatment. The RNA-seq data were reliable because the qRT–PCR results were highly similar to the RNA-seq results, which have been submitted for review.

### 4.6. Statistical Analysis

Analysis of variance (ANOVA) was performed using Statistix 8.0 (Analytical Software, Tallahassee, FL, USA). According to the ANOVA results, a significant difference was detected between the watering treatments. The means of the three treatments were then compared by the least significant difference (LSD) test at *p* < 0.05. A correlation analysis of the relative values (ratio of the value obtained with the DS/DS-R treatment to the value obtained with the CK treatment) of leaf succulence and phytohormones was performed using SPSS 22.0 (SPSS Inc., Chicago, IL, USA). The significance of the correlation coefficient was tested using Student’s t test (two-tailed). A t test was used to determine whether the correlation coefficient is significantly different from zero under the hypothesis that *ρ* = 0. The relevancies were visualized using a heatmap with R statistical software (ver. 3.4.1 Analytical Software, Tallahassee, FL, USA).

## 5. Conclusions

Leaf succulence of highly drought-tolerant *I. difengpi* plants shows marked changes in response to severe drought and rehydration. The responses of ABA, MeJA, SAG, and cZR were highly correlated with the changes in leaf succulence and the restorative effects of the exogenous application of ABA and ZR on leaf succulence under drought conditions together supported the regulatory role of phytohormones in the drought response of *I. difengpi* plants. And the key genes involved in signal transduction of ABA and JA were identified by transcriptomics profiling, which provided preliminary clues to explain the reason for changes in phytohormones induced by drought stress. Future research is needed to elucidate the molecular mechanisms of phytohormones signal transduction in *I. difengpi* plants in response to drought stress.

## Figures and Tables

**Table 1:** Effects of water treatments on concentration of phytohormones of *I. difengpi* plants.

Treatments	ABA	ACC	IAA	MeJA	SA	SAG	cZ	cZR	iP	iPA
CK	176.3 ± 6.76 ^c^	10.6 ± 0.86 ^b^	0.83 ± 0.06 ^b^	0.42 ± 0.08 ^b^	0.99 ± 0.68 ^a^	65.38 ± 4.85 ^a^	0.03 ± 0.01 ^a^	1.3 ± 0.10 ^a^	0.03 ± 0.01 ^a^	0.19 ± 0.02 ^b^
DS	704.5 ± 32.81 ^b^	12.5 ± 0.81 ^a^	0.86 ± 0.09 ^b^	0.25 ± 0.02 ^c^	0.46 ± 0.36 ^a^	47.95 ± 1.2 ^b^	0.02 ± 0.01 ^b^	0.61 ± 0.04 ^c^	0.03 ± 0.01 ^a^	0.12 ± 0.03 ^c^
DS-R	1004.2 ± 22.0 ^a^	9.8 ± 1.05 ^b^	1.04 ± 0.08 ^a^	0.86 ± 0.03 ^a^	0.66 ± 0.11 ^a^	60.58 ± 2.66 ^a^	0.01 ± 0.01 ^c^	0.78 ± 0.05 ^b^	0.01 ± 0.01 ^b^	0.24 ± 0.02 ^a^
F	1320.5 **	9.13 **	7.85 *	165.1 **	0.95	30.3 **	21.0 **	106.5 **	8.60 **	21.4 **

The data are presented as the mean ± SD (n = 4). * and ** indicate significance at *p* < 0.05 and *p* < 0.01 level, respectively. Different letters indicate significant differences among the three water treatments for the *I. difengpi* plants at the *p* < 0.05 level by a least significant difference test. ABA, abscisic acid; IAA, indole-3-acetic acid; iP, N6-isopentenyladenine; iPA, isopentenyl adenosine; SA, salicylic acid; MeJA, methyl jasmonate; SAG, salicylic acid glucoside; ACC, 1-Aminocyclopropanecarboxylic acid; cZR, cis-zeatin riboside; cZ, cis-zeatin; CK, well-watered treatment; DS, drought stress treatment; DS-R, drought-rehydration treatment.

**Table 2:** Soil moisture control under the CK, DS, and DS-R treatments.

Treatments	Dry Soil Weight (kg)	Weight of Plant (kg)	Water Supplement (kg)		Total Bowl Weight (kg)	
			0~15 days	15~18 days	0~15 days	15~18 days
CK	1.35	0.05	0.675	0.675	2.075	2.075
DS	1.35	0.05	0.135	0.135	1.535	1.535
DS-R	1.35	0.05	0.135	0.675	1.535	2.075

CK, well-watered treatment; DS, drought stress treatment; DS-R, drought-rehydration treatment.

## Data Availability

The datasets presented in this study can be found in the NCBI BioProject online repository (https://www.ncbi.nlm.nih.gov/bioproject/983054, accessed on 13 August 2023).
